# Humic acid and grafting as sustainable agronomic practices for increased growth and secondary metabolism in cucumber subjected to salt stress

**DOI:** 10.1038/s41598-024-66677-8

**Published:** 2024-07-10

**Authors:** Masoomeh Amerian, Amir Palangi, Gholamreza Gohari, Georgia Ntatsi

**Affiliations:** 1 Department of Horticultural Sciences and Engineering, Faculty of Agricultural Sciences and Engineering, Campus of Agriculture and Natural Resources, Razi University, Kermanshah, Iran; 2 Department of Horticultural Sciecne, Faculty of Agriculture, University of Maragheh, Maragheh, Iran; 3 Department of Crop Science, Laboratory of Vegetable Crops, Agricultural University of Athens, Athens, Greece

**Keywords:** Rootstock, Bio stimulants, Humic acids, Antioxidant enzymes, Hole insertion grafting, Phenolic acids, Salt stress, Plant sciences, Plant physiology

## Abstract

Salinity stress poses a significant treat to crop yields and product quality worldwide. Application of a humic acid bio stimulant and grafting onto tolerant rootstocks can both be considered sustainable agronomic practices that can effectively ameliorate the negative effects of salinity stress. This study aimed to assess the above mentioned ameliorative effects of both practices on cucumber plants subjected to saline environments. To attain this goal a factorial experiment was carried out in the form of a completely randomized design with three replications. The three factors considered were (a) three different salinity levels (0, 5, and 10 dS m^−1^ of NaCl), (b) foliar application of humic acid at three levels (0, 100, and 200 mg L^−1^), and (c) both grafted and ungrafted plants. Vegetative traits including plant height, fresh and dry weight and number of leaf exhibited a significant decrease under increasing salinity stress. However, the application of humic acid at both levels mitigated these effects compared to control plants. The reduction in relative water content (RWC) of the leaf caused by salinity, was compensated by the application of humic acid and grafting. Thus, the highest RWC (86.65%) was observed in grafting plants with 0 dS m^−1^ of NaCl and 20 mg L^−1^ of humic acid. Electrolyte leakage (EL) increased under salinity stress, but the application of humic acid and grafting improved this trait and the lowest amount of EL (26.95%) was in grafting plants with 0 dS m^−1^ of NaCl and 20 mg L^−1^ of humic acid. The highest amount of catalase (0.53 mmol H_2_O_2_ g^−1^ fw min^−1^) and peroxidase (12.290 mmol H_2_O_2_ g^−1^ fw min^−1^) enzymes were observed in the treatment of 10 dS m^−1^ of NaCl and 200 mg L^−1^ humic acid. The highest amount of total phenol (1.99 mg g^−1^ FW), total flavonoid (0.486 mg g^−1^ FW), total soluble carbohydrate (30.80 mg g^−1^ FW), soluble protein (34.56 mg g^−1^ FW), proline (3.86 µg g^−1^ FW) was in grafting plants with 0 dS m^−1^ of NaCl and 200 mg L^−1^ of humic acid. Phenolic acids and phenylalanine ammonia lyase (PAL) and polyphenol oxidase (PPO) enzymes increased with increasing salinity and humic acid levels. Contrary to humic acid, salt stress increased the sodium (Na^+^) and chlorine (Cl^−^) and decreased the amount of potassium (K^+^) and calcium (Ca^2+^) in the root and leaf of ungrafted cucumber. However, the application 200 mg L^−1^ humic acid appeared to mitigate these effects, thereby suggesting a potential role in moderating physiological processes and improving growth of cucumber plants subjected to salinity stress. According to the obtained results, spraying of humic acid (200 mg L^−1^) and the use of salt resistant rootstocks are recommended to increase tolerance to salt stress in cucumber. These results, for the first time, clearly demonstrated that fig leaf gourd a new highly salt-tolerant rootstock, enhances salt tolerance and improves yield and quality of grafted cucumber plants by reducing sodium transport to the shoot and increasing the amount of compatible osmolytes.

## Introduction

Cucumber (*Cucumis sativus* L.) is an important vegetable all over the world. Its high susceptibility to salt stress, attributed to its shallow roots, results in reduced growth and yield^1^. Similar to other salt sensitive plants, in cucumber, salinity stress leads to reduced root growth and water absorption, chlorosis and withering of leaves, and in severe stress, plant death. The evaluation of extent of these effects can an indicator for investigating salt tolerance of cucumber cultivars^2^. Salinity is one of the most important abiotic stresses that reduce plant growth and production worldwide, particularly in arid and semi-arid regions where inadequate rainfall and high soil salt concentrations occur^3,4^. Sodium (Na^+^), a key cation highly associated with salinity stress, is present in many soils of those regions, and its accumulation in plant tissues can cause nutrient imbalances, while disrupting cellular processes. Indeed, under salinity stress, the high amount of Na^+^ and Cl^−^ accumulation can disrupt Ca^2+^ and K^+^ absorption, while the accumulation of Na^+^ in plant tissues can disrupt or even destroy cellular homeostasis. To cope with salinity stress, plants tend to reduce the amount of Na^+^ in their xylem, and as a result, the accumulation of Na^+^ in their tissues is reduced^5^.

In addition, secondary stresses, such as oxidative stress are often associated with osmotic stress and ion toxicity, which are harmful to plant cells due to the accumulation of reactive oxygen species (ROS)^6^. Reactive oxygen species are inevitably generated in the reduction and oxidation reactions of plants, including respiration and photosynthesis. In parallel with the production of ROS, an advanced antioxidant defense system has evolved in aerobic organs. This system comprises antioxidant enzymes such as superoxide dismutase (SOD), ascorbate peroxidase (APX), catalase (CAT), peroxidase (POX) monodehydroascorbate reductase (NADH), dehydroascorbate reductase (DHAR), glutathione reductase (GR), glutathione peroxidase (GPX), glutathione S-transferase (GST) and guaiacol peroxidase (GOPX) and non-enzymatic compounds such as ascorbic acid (ASA), glutathione (GSH), tocopherols, phenolic compounds and non-protein amino acids. However, any disturbance in the balance between ROS and the antioxidant defense system can create conditions of oxidative stress. Reactive oxygen species can significantly damage membrane lipids, proteins, nucleic acids, and photosynthetic pigments^5^. Considerable efforts have been made to increase salt stress tolerance in plants, including the application of exogenous substances, such as compatible solutes^7^, trehalose^8^, brassinolide^9,10^, melatonin^11^, humic acid^12^ and selenium^13^. The use of organic acids to improve the quality and quantity of agricultural and horticultural crops has gained popularity. The presence of hormonal compounds in very small amounts of organic acids can significantly improve the physical, chemical and biological characteristics of the soil and concomitantly increase yield and product quality^14^.

Humic acid is a natural organic polymer compound derived from the decomposition of soil organic matter, peat, lignin, etc. One of the important benefit of humic acid is the chelation of various nutrients such as Na^+^, K^+^, magnesium (Mg^2+^), zinc, Ca^2+^, iron (Fe^2+^), copper (Cu^2+^), etc., to overcome nutrient deficiencies and increase root length, weight, and lateral root formation^15^. By modifying soil physical properties, humic acid enhances water retention by creating larger pore spaces. In addition, humic acid molecules form a bond with water molecules that prevents water evaporation. Also, humic acid increases the photosynthetic activity of the plant by increasing the activity of Rubisco enzyme^16^. Humic substances exhibit anti-stress effects under non-living stress conditions. These substances may also increase the absorption of nutrients and reduce the toxicity of some absorbed elements. Therefore, the application of humic substances, when plants are subjected to salinity stress can result in improved plant growth^17^. Furthermore, humic acid affects the activity of enzymes and secondary plant^17^.

In recent decades, various agronomic strategies have been developed to facilitate the utilization of highly saline soils without compromising agricultural productivity. These include breeding genotypes with high resilience to salt stress. However, this is a difficult and complex process because plant resistance to salt stress is a polygenic trait^18^. Nowadays, grafting have emerged as an ecofriendly alternative, where both rootstock and scion contributes to salt tolerance of grafted plants^19,20^. Grafting has also been shown to increase abiotic stress (drought, low temperature and ionic toxicity) tolerance and yield^21,22^. It is well documented that grafting can cause hormonal imbalances in the scion leading to alterations in water and nutrient accumulation^23,24^. Compared to ungrafted plants, grafted plants exhibit increased water content and photosynthesis thereby leading to increased biomass and plant yield^25,26^. The importance of root system and root characteristics in regulating salt tolerance in potato (*Solanum tuberosum* L.)^27^, pepper (*Capsicum annuum* L.)^28^ and tomato (*Solanum lycopersicum* L.)^20^ have been reported. Therefore, using genotypes resistant to salinity stress as a rootstock is a simple and efficient method to improve the product's resilience to this stress^29^. Grafting has been shown to reduce the absorption of Na^+^ by the roots and increase the amount of K^+^ in the leaf, thereby maintaining a balanced Na^+^ and K^+^ leaf ratio.

Grafting has a positive effect on osmotic regulation, enhances antioxidant enzymes activity, and improves the synthesis of compatible substances and plant tolerance against salt stress^1^. Although grafting has been widely applied in cucumber cultivation^30^, to our knowledge the impact of grafting and humic acid on cucumber salt stress tolerance has not been studied. Previously, other studies were carried out to determine the contribution of grafting to several abiotic stress tolerance mechanisms of many plant species. However, no comprehensive studies were found in the literature with regard to the salinity problem of cucumber plants. The present study is based on the hypothesis that the foliar application of humic acid at adequate concentrations and grafting mitigates the deleterious effects caused by irrigation with saline water on the growth and physiology of the cucumber, inducing plant tolerance to salt stress caused by the increase in the biosynthesis of photosynthetic pigments and the photochemical efficiency, reflecting on higher plant growth. Therefore, the purpose of this research is to investigate the tolerance of grafted cucumber under salinity stress conditions with the application of external humic acid and more comprehensive studies on the cucumber plants have to be conducted to better understand whether grafting could improve salinity tolerance.

## Materials and methods

### Site description and experiment design

The study was carried out in a greenhouse at Razi University in Kermanshah, Iran. Cucumber (*Cucumis sativus* L.) of the Negeen variety as a scion and fig leaf gourd (*Cucurbita ficifolia* L.) was used as a rootstock. seeds were obtained from the Fardin Kesht Company, alborz, Iran. In this study, the experimental treatments were implemented in a factorial design, based on completely randomized design with three replications. The first factor consisted of different salinity levels namely 0, 5, and 10 dS m^−1^ of NaCl; the second factor was the foliar application of humic acid at three levels of 0, 100, and 200 mg L^−1^, and the third factor included both grafted and ungrafted plants. There was one plant in each pot, so we had 54 plants (pots).

**Figure 1:**
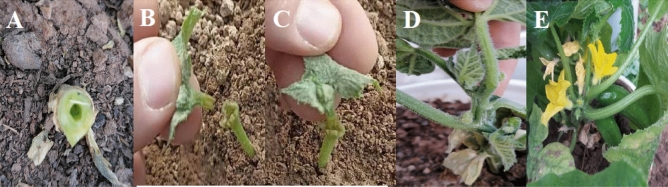
The steps to perform the grafting, preparation of the rootstock (**A**), preparation of scion (**B**), placing the scion on the rootstock (**C**), fusion of the graft site (**D**) and fruit formation on grafted cucumber (**E**).

Cucumber scion was grafting on fig leaf gourd rootstock by hole insertion grafting. cucumber seeds were sown 3 days prior to fig gourd seeds. Fig gourd seed germinates earlier, so cucumber seed was planted earlier. Soil, sand, and decomposed manure rate 1:1:1 (v/v) was used as growing media. The cucumber scions of 13 days and fig gourd rootstocks of 10 days were best match for grafting. The procedure for hole insertion grafting is demonstrated in Fig. 1. First, true leaves and meristem tissue are removed at the growing tip of the rootstock. Next, a slit is made across the growing point from the bottom of one cotyledon to the other side of the hypocotyl. A shaved stick such as a toothpick or bamboo barbecue skewer can be used as the insertion tool. Leave the stick inserted in the growing point, while cutting the scion hypocotyl at both sides into a V shape. The scion is then inserted into the slit while the stick is removed. Hole insertion grafting produces high-quality grafted transplants because it may help increase the contacting surface area between rootstock and scion and provide protection of the graft union with both rootstock cotyledons. Another advantage of this method is that it does not require grafting clips, which reduces the grafting cost as well as the labor involved in collecting clips after healing.

The plastic pots were enveloped with transparent plastic sheet in order to maintain the humidity level around the graft. The plastic sheet was cut from top side after 3 days in order to allow vertical growth and plastic sheet was taken off completely after 6 days. Once the grafted plants had developed three true leaves, salinity stress was applied by adding NaCl (Sodium chloride; Merck-India) to the irrigation water to the end of the growth period. The salinity level increased gradually to reach the desired stress level. Humic acid (Humic acid sodium salt), purchased from Sigma Company (CITY; COUNTRY), was applied by foliar spraying once when the stress was initiated. Pest and disease control, plant pruning and support for vertical growth, irrigation, and temperature and humidity regulation were all provided throughout the experiment. The environmental conditions of the greenhouse during the cucumber growth period included a day temperature of 22–26 °C and the night temperatures of 18–20 °C, light intensity 6000–10,000 Lux and relative humidity be-tween 50 and 60%. The duration of the experiment was 4 months.

### Morphometric parameters

The morphological characteristics studied were plant height, fresh weight of root, shoot and fruit, number of leaf and fruit and single plant yield (g). Fruits were harvested three times per week between December 28 and February 20. At each harvest, the total fruit number and single plant yield was recorded separately and finally mean fruit weight and total fruit yield were calculated.

To measure the firmness of the fruit tissue, the firmness tester model XTPlus-TA was used. The length and width of the fruit were measured using a digital caliper model NO: Z 22855. TSS were measured by placing a few drops of cucumber fruit extract on the ATAGO hand-held refractometer (MODEL, COMPANY, COUNTRY), and the corresponding number was read from the graduated column. To measure the fruit dry matter content, 5 g of fresh fruit from each replicate were wrapped in aluminum foil and placed in an oven at 70 °C for 48 h to constant weight dry matter content was calculated using the following relationship:

$${\text{Dry}}\;{\text{matter}}\;{\text{content}}\; (\% ) = \left( {\frac{{{\text{Dry }}\;{\text{weight}}}}{{{\text{Initial }}\;{\text{weight}}}}} \right) \times 100$$

### Physiologic parameters

Two weeks after foliar spraying with humic acid, leaf samples were transferred to the laboratory to measure physiological traits.

#### Photosynthetic pigments

Leaf photosynthetic pigments were measured according to the method of Lichtenthaler^31^. Concisely, 0.5 g of fresh leaf was weighted and ground in 10 mL 80% acetone. The obtained homogenate was centrifuged (6000 rpm) for 10 min and then the absorbance of the samples was read at 645 and 663 nm using the spectrophotometric method (Kerry 100 model, Varian, America). The amount of chlorophyll was calculated in mg FW^−1^ using the following formulas:

$${\text{Chla}}~\;({\text{mg}}\;{\text{L}}^{{ - 1}} ) = (12.7 \times {\text{A}}663) - (2.69 \times {\text{A}}645)$$

$${\text{Chlb}}\;~({\text{mg}}\;{\text{L}}^{{ - 1}} ) = (25.8 \times {\text{A}}645) - (4.68 \times {\text{A}}663)$$

$${\text{Chltotal}}\;~({\text{mg}}\;{\text{L}}^{{ - 1}} ) = (20.21 \times {\text{A}}645) + (8.02 \times {\text{A}}633)$$

#### Relative water content (RWC)

To calculate the RWC, the last developed leaf from each plant was collected and quickly transferred to the laboratory on ice cubes to measure fresh weight (FW). Concomitantly, the leaf samples were placed in a laboratory beaker containing cool distilled water for 24 h and then their turgid weight (TW) measured. To measure dry weight (DW), leaf discs were dried in an oven (120 L smart model, BF 120 S, Iran) for 48 h at 72 °C and then weighed. The leaf RWC was calculated as following formula^32^:

$${\text{RWC}}\% = \frac{{({\text{FW}} - {\text{DW}})}}{{({\text{TW}} - {\text{DW}})}} \times 100$$

#### Electrolyte leakage (EL)

Initially, 0.2 g of healthy and fresh washed leaf tissue immersed in 40 mL of deionized water and placed in tubes. The tubes were then immediately shaken (120 rpm) for 12 h at room temperature. The electrical conductivity of the samples (EC_1_) was measured with an EC meter (Milwaukee Pen EC Meter). Subsequently, the samples were autoclaved at 121 °C for 20 min, and after reaching a temperature of 25 °C, the electrical conductivity of the samples (EC_2_) was measured again.

The electrolyte leakage percentage was calculated following the equation^33^:

$${\text{EL}} = \frac{{{\text{EC}}1}}{{{\text{EC}}2}} \times 100.$$

#### Total soluble carbohydrates and proline

For total soluble carbohydrates analysis, leaf tissue (1.0 g) was grounded in 5 mL ethanol (80%; v/v) and centrifuged (10,000 rpm) for 15 min. Then 0.1 mL of the alcoholic extract was reacted with 3 mL of freshly prepared anthrone containing 150 mg of anthrone + 100 mL of 72% sulfuric acid. It was placed in a boiling water bath for 10 min. At this time, a colored substance was formed. Then cooled down immediately in ice bath to 23 °C. Glucose standards were prepared from 0 to 0.1 μmol mL^−1^. Finally, the light absorption of standard solutions and samples was read with a spectrophotometer (Kerry 100 model, Varian, America) at a wavelength of 625 nm^34^.

For proline assay, frozen leaf (0.5 g) was first ground using 5 mL ethanol (95%) in a ceramic mortar and the upper solution was separated, and its sediments were washed twice with 5 mL of 70% ethanol, and their upper phase was added to the previously collected supernatant. The obtained solution was centrifuged at 3500 rpm for 10 min. After separating the liquid and solid phases, the liquid part was kept inside the refrigerator at a temperature of 4 °C. Then 1 mL of the above-mentioned alcoholic extract was diluted with 10 mL of distilled water, and 5 mL of Ninhydrin reagent was added to it. The composition of the Ninhydrin reagent for each sample included 0.125 g of Ninhydrin + 2 mL of 6 M Phosphoric acid + 3 mL of Glacial acetic acid. After adding the Ninhydrin reagent, 5 mL of Glacial acid was added, and the resulting mixture was placed in a boiling water bath at 100 °C for 45 min. After removing the samples from the boiling water bath and cooling them, 10 mL of benzene was added to each sample and shaken vigorously until proline entered the benzene phase. The samples were then left to stand still for 30 min. Finally, the light absorption of standard solutions and samples was measured at a wavelength of 515 nm with a spectrophotometer (Kerry 100 model, Varian, America)^35^.

#### Total phenol and flavonoid

Singleton and Rossi^36^ method was used to measure total phenol. To prepare methanolic extract, 0.5 g fresh tissue of the leaf was crushed well in a mortar in the presence of 3 mL of 85% methanol and then smoothed. This methanolic extract was used to measure total phenol and flavonoids. In this method, 300 µL of methanolic extract was mixed with 1500 µL of diluted folin solution (10:1 ratio with distilled water). After keeping it for 8 min at 25 °C, 1200 µL of 7% sodium bicarbonate solution were added. After 90 min of shaking on a shaker at a speed of 120 rpm at room temperature and in the dark, the absorbance of the samples was measured with a spectrophotometer at a wavelength of 765 nm (model Kerry 100, Varian, America). Phenolic content (represented as mg g^−1^ FW) was determined based on the standard curve of gallic acid.

Total flavonoid measurement was done according to Bor et al.^37^ method. 50 µL of methanol extract was mixed with 10 µL of aluminum chloride (AlCl_3_) (10%), 10 µL of potassium acetate (1 M), and 280 µL of deionized water. After vortexing, the samples were kept at room temperature for 40 min. Finally, the samples absorbance was read spectrophotometerically at 415 nm and flavonoids content (mg g^−1^ FW) was determined based on the standard curve of quercetin.

#### Total soluble protein and antioxidant enzyme activities

The total soluble protein was measured based on Bradford^38^ method using albumin as a standard. The total soluble protein absorbance was recorded at 595 nm through spectrophotometer (Kerry 100 model, Varian, America) and its concentration presented as mg g^−1^ FW.

To determine the activity of the CAT enzyme, the frozen leaf tissue was first ground in a mortar in the presence of liquid nitrogen, and 0.1 g of it was added to a plastic tube containing 1 mL of extraction buffer and mixed. The sample was passed through a strainer, and the prepared extract was centrifuged for 15 min at a speed of 10,000 rpm at a temperature of 4 °C and the clear supernatant solution was slowly separated.  Fifty µL of plant extract was mixed with 3 mL of extraction buffer containing 50 mM sodium phosphate (pH 7.8) and 2 mM ethylenediaminetetraacetic acid, and the reaction of CAT enzyme was started by adding 5 µL of 30% hydrogen peroxide to this mixture. The changes in optical absorption of the samples were recorded at a wavelength of 240 nm for 10 min. The amount of enzyme activity was expressed as units per mg of leaf protein. Each unit of CAT activity was considered as the 1.0 mL enzyme that reduces^39^ 1.0 μmol H_2_O_2_ min^−1^.

POX enzyme activity was measured by spectrophotometry^40^. The first, 3 mL of extraction buffer (50 mM sodium phosphate (pH 7.8) and 2 mM ethylenediaminetetraacetic acid (EDTA) was poured into both control and sample cuvettes to start the peroxidase enzyme reaction. 5 µL of 30% hydrogen peroxide and 5 µL of glycol were added to them. These two cuvettes were placed in the spectrophotometer, and the number read became 0. Then, 50 µL of plant extract were added to the sample cuvette, and the changes in light absorption of the samples at 465 nm wavelength, which indicates the degree of degradation and decrease in H_2_O_2_ concentration, were recorded every 10 s for 120 s. Each unit of peroxidase enzyme activity was considered as the amount of enzyme that reduces 1 µL of H_2_O_2_ mL^−1^ min^−1^.

#### Extraction and determination of phenolic acids

To determine the dynamics of phenolic acids, leaves were ground in liquid nitrogen. Approximately 1 g of the frozen powder was mixed with 3 mL of methanol. The extract was centrifuged at 14,000 rpm for 15 min. The pellet was re-suspended in 3 mL of methanol and re-extracted at 14,000 rpm for 15 min. Supernatants from both extractions were combined and dried under N_2_ at room temperature. The residue was re-suspended in 3 mL of water at 80 °C for 10 min. The solution was then split into two proportions. One proportion (1 mL) for measuring the content of free phenolic acids was extracted with a mixture, containing 2.5 mL of cyclohexane/ethyl acetate (1:1, v/v) and 50 µL of HCl. The other proportion (1 mL) was used for measuring phenolic acids. For this measurement 1 mL of 50 mM sodium acetate (pH 4.5) containing β-glucosidase was added to the sample which was then incubated at 37 °C for 6 h and extracted with the same mixture as mentioned above. The organic phase was removed and dried under N_2_ at room temperature. The residue was dissolved in 200 µL of methanol. The supernatant was filtered through 0.45 µm nylon filters prior to HPLC (Unicam-Crystal-200) analysis^41^.

#### Determination of phenylalanine ammonia lyase (PAL) and polyphenol oxidase (PPO) activities

To determine the changes of PAL activity, 0.3 g of plant tissue was extracted with 2 mL of 50 mM boracic acid buffer (pH 8.8), containing 8 mM β-mercaptoethanol and 2% (w/v) PVPP. The homogenate was centrifuged for 20 min at 14,000 rpm. PAL assay was performed with l-phenylalanine as the substrate, at 290 nm^42^.

The enzyme extract of PPO was carried out as described by Lamikanra and Watson^43^. After centrifugation, PPO activity was assayed following the method of Luh and Phithakpol^44^ in the wave length 398 nm.

All operations were carried out at 0–4 °C. All spectrophotometric analyses were conducted on a Kerry 100 model, Varian, USA, spectrophotometer.

### Leaf mineral concentrations

In order to extract and measure the concentration of K^+^, Ca^2+^, Na^+^ and Cl^−^ healthy leaf samples were washed with distilled water. Samples were dried and grounded at 75 °C for 72 h. After preparation of the extract by wet digestion method using concentrated nitric acid (65%), the concentration of K^+^ and Na^+^ was measured using a flame photometer (model G 405, Crouse company, Germany) while Ca^2+^ detected by an atomic absorption spectrometer (Kerry 100 model, Varian, America). To measure Cl^−^, 100 mg of plant tissue was pulverized and poured into a Falcon tube. Extraction was performed after adding 10 mL of 0.5 M nitric acid and placing it for one hour at 80 °C. 1 mL of the extract was used to read the Cl^−^ concentration according to the colorimetric ferricyanide method at 480 nm^45^.

### Statistical analyses

Data were analyzed with SAS (9.1) statistical software. Mean comparisons were performed with Duncan’s multiple range test at the 5% level of significance. The experimental treatments were implemented in a factorial design, based on completely randomized design with three replications The first factor consisted of different salinity levels namely 0, 5, and 10 dS m^−1^ of NaCl; the second factor was the foliar application of humic acid at three levels of 0, 100, and 200 mg L^−1^ of humic acid, and the third factor included both grafted and ungrafted plants.

### Ethical approval

It is certified that the author has complied with ethical requirements. Plant materials and testing have been done according to rules and regulations.

## Results

### Morphometric parameters

Based on our results, salt stress, humic acid, grafting and their interaction had a significant effect (p ≤ 0.01) on the growth characteristics of cucumber (Table 1). The growth characteristics of cucumber, including plant height, fresh and dry weight of root and stem and number of leaf, were significantly affected by the different salinity levels (Table 1). Salt stress significantly reduced cucumber growth compared to the control. However, under salinity stress conditions the application of humic acid and grafting significantly enhanced the growth characteristics of cucumber (Table 2). The most favorable conditions for cucumber growth increase were observed in the treatment involving grafted cucumber grown under 0 dS m^−1^ NaCl and supplemented with 200 mg L^−1^ of humic acid.

**Table 1:** Results of analysis of variance (ANOVA) effect different levels of salt stress, humic acid and grafting on plant height, fresh and dry shoot and root weight and number of leaf.

Means of square							
S.O.V	df	Plant height	Fresh stem weight	Dry stem weight	Fresh root weight	Dry root weight	Number of leaf
Salt stress	2	51,237.55**	42,988.19**	3487.19**	449.24**	19.41**	2156.51**
Humic acid	2	6969.66**	66,506.38**	585.99**	45.48**	4.55**	232.21**
Grafting	1	1686.72**	13,987.68**	144.38**	16.55**	0.93**	46.85**
Salt stress × humic acid	4	517.75**	5478.43**	34.45**	1.49**	0.88**	19.04**
Salt stress × grafting	2	246.79*	281.69^ns^	7.54^ns^	1.55**	0.153**	7.28**
Humic acid × grafting	2	165.95^ns^	721.71*	0.1451^ns^	0.7012*	0.0068^ns^	2.06*
Salt stress × humic acid × grafting	4	233.69*	500.99*	6.92*	0.71**	0.06*	3.07**
Error	36	82.82	196.96	6.77	0.1755	0.0274	0.56
C.V (%)	–	4.16	3.18	4.18	1.68	2.74	1.83

*ns* non-significant effect; *significant effect at the 0.05 level and **significant effect at the 0.01 level.

**Table 2:** Effect of different levels of salt stress, humic acid and grafting on plant height, fresh and dry shoot and root weight and number of leaf.

Salt stress (dS m^−1^)	Humic acid (mg L^−1^)	Grafting	Plant height (cm)	Fresh stem weight (g)	Dry stem weight (g)	Fresh root weight (g)	Dry root weight (g)	Number of leaf (–)
0	0	Ungrafting	240.36 ± 1.76^de^	511.53 ± 1.86^e^	69.26 ± 0.37^cd^	27.13 ± 0.06^d^	6.50 ± 0.05^ef^	47.70 ± 0.72^e^
		Grafting	254.55 ± 5.44^cd^	521.43 ± 2.33^e^	71.47 ± 0.30^c^	28.42 ± 0.20^c^	6.70 ± 0.03^de^	49.35 ± 0.08^d^
	100	Ungrafting	270.10 ± 0.79^bc^	547.40 ± 8.02^d^	75.80 ± 1.37^b^	29.10 ± 0.13^c^	6.85 ± 0.03^cd^	50.05 ± 0.15^d^
		Grafting	276.10 ± 1.78^b^	584.53 ± 16.27^c^	77.40 ± 0.21^b^	30.10 ± 0.10^b^	7.03 ± 0.06^bc^	51.40 ± 0.10^c^
	200	Ungrafting	284.13 ± 2.68^b^	662.03 ± 9.39^b^	80.03 ± 0.3^b^	30.43 ± 0.08^b^	7.30 ± 0.10^b^	53.33 ± 0.59^b^
		Grafting	327.13 ± 21.17^a^	729.90 ± 18.08^a^	84.87 ± 1.68^a^	33.36 ± 0.85^a^	7.80 ± 0.17^a^	55.43 ± 0.39^a^
5	0	Ungrafting	195.36 ± 2.29^hi^	387.00 ± 6.62^i^	58.46 ± 0.29^ghi^	23.23 ± 0.27^h^	5.93 ± 0.03^hij^	39.90 ± 0.15^h^
		Grafting	199.73 ± 0.54^hi^	420.70 ± 8.02^h^	59.26 ± 0.22^ghi^	24.36 ± 0.06^g^	6.00 ± 0.00^ghi^	40.33 ± 0.14^h^
	100	Ungrafting	204.10 ± 0.82^gh^	439.40 ± 5.53^gh^	60.16 ± 0.16^fgh^	24.80 ± 0.05^fg^	6.10 ± 0.00^ghi^	41.76 ± 0.29^g^
		Grafting	215.30 ± 1.23^fg^	455.20 ± 2.83^fg^	63.06 ± 0.40^efg^	25.130 ± 0.14^f^	6.130 ± 0.03^ghi^	42.60 ± 0.10^g^
	200	Ungrafting	226.63 ± 0.49^ef^	475.63 ± 8.41^f^	64.06 ± 0.23^ef^	25.83 ± 0.12^e^	6.20 ± 0.00^gh^	44.40 ± 0.32^f^
		Grafting	231.10 ± 0.89^ef^	500.67 ± 2.37^e^	66.33 ± 1.06^de^	26.20 ± 0.05^e^	6.30 ± 0.00^fg^	45.13 ± 0.13^f^
10	0	Ungrafting	152.26 ± 1.70^m^	219.47 ± 8.35^n^	36.80 ± 5.81^l^	17.90 ± 0.05^l^	3.66 ± 0.27^n^	20.60 ± 0.30^n^
		Grafting	159.80 ± 1.07^m^	244.87 ± 2.94^m^	43.86 ± .0.47^k^	18.43 ± 0.08^l^	4.33 ± 0.03^m^	26.43 ± 0.73^m^
	100	Ungrafting	163.80 ± 0.97^lm^	274.70 ± 8.82^l^	46.53 ± 1.09^k^	19.23 ± 0.14^k^	4.86 ± 0.18^l^	26.43 ± 0.78^l^
		Grafting	170.46 ± 1.38^jkl^	295.97 ± 3.10^k^	51.06 ± 0.58^j^	20.20 ± 0.10^j^	5.30 ± 0.05^k^	30.96 ± 0.30^k^
	200	Ungrafting	178.46 ± 0.90^jk^	306.53 ± 1.76^k^	55.00 ± 0.00^ji^	20.63 ± 0.14^j^	5.66 ± 0.03^j^	33.40 ± 0.73^j^
		Grafting	185.50 ± 2.65^ij^	353.33 ± 8.98^j^	57.13 ± 0.30^hi^	22.00 ± 0.29^i^	5.86 ± 0.03^ij^	35.63 ± 0.54^i^

In each column means that a common letters are significantly different at the5% level (Duncan's multiple range tests). Values express mean ± SE (n = 3).

The results of variance analysis of the effect of different levels of salt stress, humic acid and grafting on the yield and yield components of cucumber showed that interaction between three factors also had a significant effect on fruit width, dry matter, firmness (p ≤ 0.01), fresh fruit weight, number of fruits, fruit length, TSS and plant yield (p ≤ 0.05) (Table 3). Based on the obtained results, the yield and yield components of cucumber decreased with increase in salinity levels. However, humic acid application resulted in improved fruit fresh weight, fruit number, dry matter content, firmness, TSS and single plant yield of grafted cucumber. Thus, the treatment of 0 dS m^−1^ NaCl with 200 mg L^−1^ of humic acid in grafted cucumber produced the highest yield and yield components (Table 4). Conversely, the lowest yield and yield components were observed in ungrafted cucumber grown at 10 dS m^−1^ NaCl and 0 mg L^−1^ of humic acid were applied (Table 4).

**Table 3:** Results of analysis of variance (ANOVA) effect different levels of salt stress, humic acid and grafting on fresh fruit weight, number of fruits, fruit length, fruit width, dry matter, firmness, TSS and plant yield.

Means of square									
S.O.V	df	Fresh fruit weight	Number of fruits	Fruit width	Fruit length	Dry matter	Firmness	TSS	Plant yield
Salt stress	2	29,299.93**	1029.58**	29.97**	53.08**	1.53**	0.539**	1.44**	40,062,121.24**
Humic acid	2	1630.14**	79.45**	4.32**	7.55**	0.105**	0.193**	0.115**	2,639,287.24**
Grafting	1	223.66**	17.22**	0.88**	1.40**	0.021**	0.042**	0.031**	466,032.85**
Salt stress × humic acid	4	40.60*	9.64**	0.033*	0.16**	0.011**	0.067**	0.022**	190,658.33**
Salt stress × grafting	2	33.87*	2.09**	0.088**	0.05**	0.0018*	0.0076*	0.006**	190,658.33*
Humic acid × grafting	2	8.49**	1.63*	0.002^ns^	0.0116^ns^	0.00023^ns^	0.0064^ns^	0.0003^ns^	17,272.90*
Salt stress × humic acid × grafting	4	35.70*	2.28*	0.13**	0.0113*	0.0028**	0.015**	0.0047*	25,624.16*
Error	36	24.37	0.66	0.009	0.016	0.00054	0.0027	0.0009	15,764.22
C.V (%)	–	4.84	4.34	2.21	0.76	1.69	3.26	1.94	2.35

*ns* non-significant effect; *significant effect at the 0.05 level and **significant effect at the 0.01 level.

**Table 4:** Effect of different levels of salt stress, humic acid and grafting on fresh fruit weight, number of fruits, fruit length, fruit width, dry matter, firmness, TSS and plant yield.

Salt stress (dS m^−1^)	Humic acid (mg L^−1^)	Grafting	Fresh fruit weight (g)	Number of fruits (–)	Fruit length (cm)	Fruit width (cm)	Dry matter (%)	Firmness (N cm^2^)	TSS (Brix)	Plant yield (g)
0	0	Ungrafting	128.00 ± 3.06^cd^	22.83 ± 0.12^ef^	16.90 ± 0.05^f^	5.02 ± 0.00^de^	3.52 ± 0.01^d^	1.69 ± 0.00^bcd^	3.50 ± 0.00^ef^	2923.1 ± 0.00^ef^
		Grafting	139.00 ± 1.85^c^	23.75 ± o.12^de^	17.15 ± 0.06^e^	5.16 ± 0.04^d^	3.58 ± 0.00^c^	1.70 ± 0.00^bc^	3.53 ± 0.01^de^	3301.7 ± 0.00^e^
	100	Ungrafting	145.99 ± 1.14^bc^	24.80 ± 0.29^cd^	17.55 ± 0.05^d^	5.50 ± 0.06^c^	3.60 ± 0.00^c^	1.71 ± 0.00^bc^	3.58 ± 0.00^cd^	3596.28 ± 0.02^d^
		Grafting	149.40 ± 0.71^b^	25.83 ± 0.33^bc^	17.86 ± 0.06^c^	5.60 ± 0.05^c^	3.61 ± 0.01^c^	1.72 ± 0.00^bc^	3.60 ± 0.00^c^	3860.0 ± 0.06^c^
	200	Ungrafting	154.00 ± 1.16^ab^	26.86 ± 0.06^ab^	18.13 ± 0.08^b^	5.90 ± 0.00^b^	3.68 ± 0.01^b^	1.78 ± 0.01^b^	3.66 ± 0.01^b^	4137.6 ± 0.07^b^
		Grafting	158.00 ± 4.81^a^	27.53 ± 0.24^a^	18.56 ± 0.06^a^	6.13 ± 0.06^a^	3.73 ± 0.02^a^	1.93 ± 0.06^a^	3.80 ± 0.05^a^	4350.8 ± 28.01^a^
5	0	Ungrafting	91.66 ± 0.77^gh^	19.26 ± 0.14^j^	15.46 ± 0.03^j^	4.00 ± 0.00^g^	3.25 ± 0.02^h^	1.58 ± 0.00^efgh^	3.17 ± 0.02^i^	1701.2 ± 0.02^j^
		Grafting	85.33 ± 2.09^h^	19.93 ± 0.23^ji^	15.66 ± 0.06^j^	4.04 ± 0.00^g^	3.31 ± 0.01^g^	1.59 ± 0.00^defgh^	3.30 ± 0.00^h^	1763.3 ± 0.00^j^
	100	Ungrafting	90.33 ± 1.48^gh^	20.50 ± 0.11^hji^	15.96 ± 0.03^i^	4.12 ± 0.04^g^	3.37 ± 0.01^f^	1.62 ± 0.00^cdefg^	3.33 ± 0.03^h^	1852.2 ± 0.05^ij^
		Grafting	96.00 ± 0.13^fg^	20.93 ± 0.12^ghi^	16.13 ± 0.03^i^	4.60 ± 0.11^f^	3.44 ± 0.02^e^	1.64 ± 0.00^cdef^	3.45 ± 0.01^g^	2009.7 ± 0.06^hi^
	200	Ungrafting	101.13 ± 3.11^ef^	21.40 ± 0.10^gh^	16.36 ± 0.06^h^	4.96 ± 0.03^e^	3.50 ± 0.00^d^	1.64 ± 0.00^cdef^	3.45 ± 0.00^fg^	2164.4 ± 0.00^gh^
		Grafting	105.03 ± 2.37^e^	22.00 ± 0.17^fg^	16.63 ± 0.06^g^	5.00 ± 0.00^de^	3.50 ± 0.00^d^	1.66 ± 0.00^cde^	3.48 ± 0.00^ef^	2311.0 ± 0.01^g^
10	0	Ungrafting	52.66 ± 1.82^l^	7.63 ± 0.08^n^	13.20 ± 0.11^p^	2.06 ± 0.01^l^	3.00 ± 0.00^k^	1.04 ± 0.01^j^	3.03 ± 0.00^j^	402.2 ± 0.06^n^
		Grafting	57.66 ± 1.58^kl^	8.30 ± 0.05^mn^	13.63 ± 0.08^o^	2.76 ± 0.08^k^	3.03 ± 0.00^jk^	1.29 ± 0.10^i^	3.03 ± 0.00^j^	478.7 ± 0.01^mn^
	100	Ungrafting	63.66 ± 0.35^jk^	8.66 ± 0.06^mn^	14.06 ± 0.06^n^	3.01 ± 0.00^j^	3.04 ± 0.00^ijk^	1.50 ± 0.00^h^	3.04 ± 0.00^j^	551.9 ± 0.02^mn^
		Grafting	69.337 ± 1.20^ij^	9.46 ± 0.24^m^	14.53 ± 0.03^m^	3.05 ± 0.00^ji^	3.06 ± 0.00^ij^	1.53 ± 0.00^gh^	3.04 ± 0.00^j^	656.9 ± 0.00^m^
	200	Ungrafting	73.00 ± 2.78^i^	12.20 ± 0.21^l^	14.80 ± 0.11^l^	3.20 ± 0.05^i^	3.07 ± 0.00^ij^	1.55 ± 0.00^fgh^	3.05 ± 0.00^j^	890.8 ± 0.01^l^
		Grafting	76.33 ± 2.22^i^	16.40 ± 1.89^k^	15.20 ± 0.11^k^	3.70 ± 0.11^h^	3.08 ± 0.00^i^	1.56 ± 0.00^fgh^	3.06 ± 0.00^j^	1256.4 ± 0.03^k^

In each column means that a common letters are significantly different at the5% level (Duncan's multiple range tests). Values express mean ± SE (n = 3).

### Physiologic parametters

#### Photosynthetic pigments

The results of variance analysis of the effect of different levels of salt stress, humic acid and grafting on photosynthetic pigments showed the interaction between salt stress and humic acid also had a significant effect on photosynthetic pigments (p ≤ 0.01) (Table 5). While the interaction between salinity, humic acid and grafting on photosynthetic pigments was not significant (Table 5). Contrary to humic acid application, salt stress decreased the concentration of chlorophyll *a*, chlorophyll *b* and total chlorophyll in cucumber leaves. With increasing salinity, untreated cucumber plants exhibited a decrease in the amount of photosynthetic pigments. However, the application of 200 mg L^−1^ humic acid increased the amount of photosynthetic pigments under salinity stress conditions (Table 6). The highest amount of chlorophyll *a* (15.76 mg g^−1^ FW), chlorophyll *b* (4.45 mg g^−1^ FW) and total chlorophyll (19.91 mg g^−1^ FW) was recorded in the in 0 dS m^−1^ NaCl treatment with 200 mg L^−1^ of humic acid, regardless of the grafting treatment (Table 6).

**Table 5:** Results of analysis of variance (ANOVA) effect different levels of salt stress, humic acid and grafting on photosynthetic pigments cucumber leaf.

Means of square				
S.O.V	df	Chlorophyll *a*	Chlorophyll *b*	Total chlorophyll
Salt stress	2	177.76**	85.76**	20.58**
Humic acid	2	28.20**	12.67**	3.20**
Grafting	1	4.95**	2.06**	0.751**
Salt stress × humic acid	4	3.45**	0.37**	0.23**
Salt Stress × grafting	2	0.52^ns^	0.081^ns^	0.041^ns^
Humic acid × grafting	2	0.022^ns^	0.096^ns^	0.038^ns^
Salt stress × humic acid × grafting	4	0.037^ns^	0.14^ns^	0.018^ns^
Error	36	0.0900	0.046	0.0091
C.V (%)	–	2.10	1.79	3.38

*ns* non-significant effect; *significant effect at the 0.05 level and **significant effect at the 0.01 level.

**Table 6:** Mean comparison of the effect of different levels of salt stress and humic acid on chlorophyll *a*, chlorophyll *b* and total chlorophyll.

Salt stress (dS m^−1^)	Humic acid (mg L^−1^)	Chlorophyll *a* (mg g^−1^ FW)	Chlorophyll *b* (mg g^−1^ FW)	Total chlorophyll (mg g^−1^ FW)
0	0	13.73 ± 0.08^c^	3.40 ± 0.03^c^	15.62 ± 0.21^c^
	100	14.13 ± 0.05^b^	3.86 ± 0.05^b^	17.57 ± 0.32^b^
	200	15.76 ± 0.31^a^	4.45 ± 0.12^a^	19.91 ± 0.30^a^
5	0	11.65 ± 0.08^f^	2.61 ± 0.02^e^	13.05 ± 0.13^f^
	100	12.28 ± 0.09^e^	2.76 ± 0.02^e^	13.90 ± 0.11^e^
	200	13.05 ± 0.06^d^	3.00 ± 0.05^d^	14.67 ± 0.07^d^
10	0	9.43 ± 0.09^i^	1.22 ± 0.06^h^	10.45 ± 0.12^i^
	100	10.14 ± 0.07^h^	1.77 ± 0.08^g^	11.28 ± 0.10^h^
	200	10.96 ± 0.09^g^	2.31 ± 0.07^f^	12.13 ± 0.07^g^

In each column means that a common letters are significantly different at the 5% level (Duncan's multiple range tests). Values express mean ± SE (n = 3).

#### Proline

The results of variance analysis of the effect of different levels of salt stress, humic acid, grafting on some physiological characteristics of cucumber showed that interaction between three factors also had a significant effect on total soluble carbohydrates, total soluble protein, total flavonoid, RWC (p ≤ 0.01), proline, total phenol and EL (p ≤ 0.05) (Table 7). However, the interaction between three factors didn't significantly effect on catalase and peroxidase (Table 7).

**Table 7:** Results of analysis of variance (ANOVA) effect different levels of salt stress, humic acid and grafting on proline, total soluble carbohydrates, total soluble protein, total phenol, total flavonoid, RWC.

Means of square										
S.O.V	df	Proline	Total soluble carbohydrates	Total soluble protein	Total phenol	Total flavonoid	RWC	EL	Catalase	Peroxidase
Salt stress	2	12.28**	419.74**	479.15**	4.01**	0.0973**	624.10**	2995.57**	0.014**	3.75**
Humic acid	2	2.40**	103.87**	149.27**	0.51**	0.00804**	147.74**	388.81**	0.009**	0.62**
Grafting	1	0.41**	11.94**	22.63**	0.06**	0.001518*	48.71**	100.01**	0.0075**	0.086**
Salt stress × humic acid	4	0.55**	38.84**	29.17**	0.04**	0.00045**	18.63**	33.43**	0.0068**	0.56**
Salt stress × grafting	2	0.05**	3.62**	3.38*	0.00007^ns^	0.000033**	11.76**	17.46**	0.0062^ns^	0.073^ns^
Humic acid × grafting	2	0.02*	1.10**	1.72^ns^	0.0019^ns^	0.000025**	5.65**	1.21^ns^	0.0061^ns^	0.0109^ns^
Salt stress × humic acid × grafting	4	0.02*	2.94**	3.86**	0.0042*	0.000040**	13.49**	2.07*	0.0061^ns^	0.0104^ns^
Error	–	0.006	0.41	0.85	0.0036	0.000022	0.569	2.03	0.0018	0.0272
C.V (%)	–	3.91	3.34	4.07	4.48	1.232	1.96	3.19	9.81	8.86

*ns* non-significant effect; *significant effect at the 0.05 level and **significant effect at the 0.01 level.

The proline content in the leaf of grafted cucumber increased with increasing salinity level, reaching the highest concentration at 10 dS m^−1^ NaCl. The application of 200 mg L^−1^ humic acid positively influenced proline content of grafted cucumber under salt stress conditions. In specific, the highest amount of proline (3.86 µg g^−1^ FW) was recorded in the concentration of 10 dS m^−1^ NaCl accompanied by the application of 200 mg L^−1^ of humic acid in grafted cucumber (Table 8).

**Table 8:** Effect of different levels of salt stress, humic acid and grafting on proline, total soluble carbohydrates, soluble protein, total phenol, total flavonoid, RWC and EL.

Salt stress (dS m^−1^)	Humic acid (mg L^−1^)	Grafting	Proline (µg g^−1^ FW)	Total soluble carbohydrates (mg g^−1^ FW)	Total soluble protein (mg g^−1^ FW)	Total phenol (mg g^−1^ FW)	Total flavonoid (mg g^−1^ FW)	RWC (%)	EL (%)
0	0	Ungrafting	1.03 ± 0.03^m^	14.13 ± 0.06^o^	13.04 ± 0.02^k^	0.59 ± 0.02^l^	0.300 ± 0.00^p^	80.98 ± 0.04^c^	38.12 ± 0.32^i^
		Grafting	1.19 ± 0.01^l^	14.70 ± 0.11^no^	16.26 ± 1.13^j^	0.61 ± 0.00^l^	0.303 ± 0.00^op^	81.22 ± 0.03^c^	35.73 ± 0.33^j^
	100	Ungrafting	1.32 ± 0.03^k^	15.15 ± 0.05^mno^	18.19 ± 0.60^i^	1.00 ± 0.12^k^	0.306 ± 0.00^op^	81.55 ± 0.03^c^	33.00 ± 0.58^k^
		Grafting	1.36 ± 0.01^jk^	15.50 ± 0.11^lmn^	19.56 ± 0.24^ih^	1.01 ± 0.01^k^	0.309 ± 0.00^no^	81.87 ± 0.06^c^	31.44 ± 0.53^kl^
	200	Ungrafting	1.41 ± 0.01^jk^	16.00 ± 0.05^klm^	20.00 ± 0.00^h^	1.11 ± 0.00^j^	0.316 ± 0.00^n^	83.87 ± 1.19^b^	29.46 ± 0.55^l^
		Grafting	1.50 ± 0.01^j^	16.40 ± 0.11^jkl^	20.06 ± 0.02^h^	1.14 ± 0.00^ij^	0.333 ± 0.00^m^	87.65 ± 0.23^a^	26.95 ± 0.29^m^
5	0	Ungrafting	1.64 ± 0.02^i^	16.80 ± 0.11^ijk^	20.81 ± 0.21^gh^	1.18 ± 0.01^hi^	0.346 ± 0.00^l^	77.40 ± 0.00^e^	46.05 ± 0.46^f^
		Grafting	1.70 ± 0.00^hi^	17.23 ± 0.06^hki^	21.28 ± 0.12^fgh^	1.22 ± 0.00^gh^	0.356 ± 0.00^k^	77.87 ± 0.23^e^	45.13 ± 0.12^f^
	100	Ungrafting	1.79 ± 0.00^h^	17.70 ± 0.11^ghi^	22.01 ± 0.00^efg^	1.26 ± 0.01^g^	0.373 ± 0.00^j^	79.87 ± 0.57^e^	43.66 ± 0.37^fg^
		Grafting	1.95 ± 0.02^g^	18.20 ± 0.11^fgh^	22.11 ± 0.04^efg^	1.31 ± 0.00^f^	0.386 ± 0.00^i^	80.45 ± 0.24^cd^	42.29 ± 0.29^gh^
	200	Ungrafting	2.02 ± 0.01^g^	18.80 ± 0.11^efg^	22.91 ± 0.10^def^	1.36 ± 0.01^f^	0.400 ± 0.00^h^	80.75 ± 0.02^c^	41.29 ± 0.11^gh^
		Grafting	2.16 ± 0.02^f^	19.13 ± 0.08^def^	23.34 ± 0.19^de^	1.46 ± 0.02^e^	0.413 ± 0.00^g^	80.79 ± 0.00^c^	40.35 ± 0.38^hi^
10	0	Ungrafting	2.22 ± 0.02^ef^	19.73 ± 0.14^de^	24.01 ± 0.00^d^	1.64 ± 0.04^d^	0.426 ± 0.00^f^	62.42 ± 0.09^h^	69.13 ± 2.47^a^
		Grafting	2.30 ± 0.00^e^	20.20 ± 0.11^cd^	24.07 ± 0.01^d^	1.75 ± 0.01^c^	0.440 ± 0.00^e^	70.87 ± 0.09^g^	62.16 ± 1.05^b^
	100	Ungrafting	2.60 ± 0.05^d^	21.16 ± 0.48^c^	24.29 ± 0.15d	1.79 ± 0.00^c^	0.453 ± 0.00^d^	71.10 ± 0.00^g^	59.33 ± 0.73^c^
		Grafting	3.06 ± 0.08^c^	25.96 ± 1.47^b^	28.46 ± 1.81^d^	1.87 ± 0.03^b^	0.466 ± 0.00^c^	71.89 ± 0.25^g^	55.96 ± 0.46^d^
	200	Ungrafting	3.50 ± 0.11^b^	30.13 ± 0.08^a^	32.73 ± 0.32^b^	1.95 ± 0.00^a^	0.476 ± 0.00^b^	75.04 ± 1.19^f^	53.69 ± 0.44^d^
		Grafting	3.86 ± 0.08^a^	30.80 ± 0.21^a^	34.56 ± 0.20^a^	1.99 ± 0.00^a^	0.486 ± 0.00^a^	79.90 ± 0.30^e^	49.32 ± 1.60^e^

In each column means that a common letters are significantly different at the5% level (Duncan's multiple range tests). Values express mean ± SE (n = 3).

#### Total soluble carbohydrates

In the present study, a significant increase in total soluble carbohydrates in grafted cucumber with the increase of NaCl was noted. Foliar spraying of humic acid at a concentration of 200 mg L^−1^ increased the amount of total soluble carbohydrates when cucumber grafted plants were subjected to 10 dS m^−1^ of NaCl (Table 8). The interaction between the three factors revealed that increasing the concentration of humic acid, the concentration of total soluble carbohydrates in grafted cucumber in all three salinity levels was increased.

#### Total soluble protein

In grafted cucumber, the amount of total soluble protein increased with increasing salinity level. Humic acid application increased the amount of total soluble protein in both grafted and ungrafted cucumber. Thus, the highest amount of total soluble protein (34.56 mg g^−1^ FW) was observed in the treatment of 200 mg L^−1^ humic acid along with 10 dS m^−1^ NaCl in grafted cucumber. In contrast, the control treatment displayed the lowest amount of total soluble protein (13.04 mg g^−1^ FW) (Table 8).

#### Total phenol

As salinity level increased, leaf total phenol of grafted cucumber increased, with the highest amount found at the concentration of 10 dS m^−1^ NaCl. Foliar application of 200 mg L^−1^ humic acid significantly increased total phenolic content of grafted cucumber leaf under salt stress conditions (Table 8). The statistical analysis of mean comparisons showed that, across all three salinity levels total phenol content increased with the increase of humic acid concentration. In specific, the highest amount of total phenol was recorded in the treatment of 10 dS m^−1^ NaCl with 200 mg L^−1^ of humic acid, while the differences between grafted and ungrafted cucumbers were insignificant.

#### Total flavonoid

The total flavonoid content of leaf of grafted cucumber increased as salinity level escalated. Across all three salinity levels, the highest total flavonoid content was observed in grafted cucumber supplemented with the highest level of humic acid (200 mg L^−1^). Specifically, the highest total flavonoid concentration (0.486 mg g^−1^ FW) was recorded in grafted cucumber subjected to 10 dS m^−1^ NaCl along with 200 mg L^−1^ of humic acid (Table 8).

#### Relative water content (RWC)

According to the obtained results, the highest amount of RWC (78/65%) was recorded in grafted cucumber treated with 200 mg L^−1^ of humic acid under salt stress conditions. In all 3 levels of NaCl, the RWC of grafted cucumber increased with the increase of humic acid concentration. Conversely, as the salinity level increased, the RWC of both grafted and ungrafted cucumber decreased (Table 8).

#### Electrolyte leakage (EL)

In contrast to salinity, humic acid decreased the rate of EL. The highest EL was observed in the treatment with 10 dS m^−1^ NaCl and 0 mg L^−1^ of acid humic in ungrafted cucumber (Table 8). The lowest EL (26.95%) was recorded in the treatment without salinity and with 200 mg L^−1^ of humic acid in grafted cucumber (Table 8).

#### Antioxidant enzymes (CAT and POX activity)

Based on the results of analysis of variance humic acid, grafting and interaction between salt stress and humic acid had a significant effect on CAT and POX (p ≤ 0.01) (Table 7). As the salinity level increased from 0 to 10 dS m^−1^ NaCl, the activity of catalase (CAT) and peroxidase (POX) increased. In addition, humic acid positively impacted the activity of CAT and POX. Consequently, the highest activity levels of CAT (0.053 mmol H_2_O_2_ g^−1^ FW min^−1^) and POX (1.29 mmol H_2_O_2_ g^−1^ FW min^−1^) were observed at the highest level of salinity and humic acid concentration (Table 9).

**Table 9:** Mean comparison of the effect of different levels of salt stress and humic acid on the activity of catalase (CAT) and peroxidase (POX) enzymes.

Salt stress (dS m^−1^)	Humic acid (mg L^−1^)	CAT (mmol H_2_O_2_ g^−1^ fw min^−1^)	POX (mmol H_2_O_2_ g^−1^ fw min^−1^)
0	0	0.020 ± 0.00^h^	0.049 ± 0.00^c^
	100	0.027 ± 0.00^g^	0.066 ± 0.00^c^
	200	0.033 ± 0.00^f^	0.074 ± 0.00^c^
5	0	0.039 ± 0.00^e^	0.079 ± 0.00^c^
	100	0.038 ± 0.00^de^	0.084 ± 0.00^c^
	20	0.040 ± 0.00^cd^	0.092 ± 0.00^c^
10	0	0.042 ± 0.00^c^	0.255 ± 0.14^b^
	100	0.045 ± 0.00^b^	1.000 ± 0.00^a^
	200	0.053 ± 0.00^a^	1.290 ± 0.12^a^

In each column means that a common letters are significantly different at the5% level (Duncan's multiple range tests). Values express mean ± SE (n = 3).

#### Phenolic acids and saponin

Based on our results, salt stress, humic acid, grafting and their interaction had a significant effect (p ≤ 0.01) on phenolic acids and saponin of cucumber (Table 10). The results of the present study clearly indicate the significant effect of salinity on phenolic acids content, with an increase in salinity leading to elevated phenolic acids concentration (Cinnamic acid, p-Coumaric acid, Caffeic acid and Ferulic acid) compared to the control. By increasing the concentration of humic acid from 0 to 200 mg L^−1^, the amount of phenolic acids in grafted cucumber increased (Table 11). Foliar spraying of humic acid had a positive effect on saponin content of grafted cucumber leaf. In all three salinity levels, the amount of saponin in grafted cucumber increased with the increase in humic acid level. The highest amount of saponin (58.66 μg g^−1^ FW) was observed in the highest concentration of salinity and humic acid in grafted cucumber (Table 11).

**Table 10:** Results of analysis of variance (ANOVA) effect different levels of salt stress, humic acid and grafting on cucumber leaf cinnamic acid, p-coumaric acid, caffeic acid, cerulic acid, saponin, phenylalanine ammonia lyase (PAL) and polyphenol oxidase (PPO).

Means of square								
S.O.V	df	Cinnamic acid	p-Coumaric acid	Caffeic acid	Saponin	Ferulic acid	PAL activity	PPO
Salt stress	2	0.1127**	8.64**	0.2632**	2559.09**	8.155**	16.46**	147.9111**
Humic acid	2	0.0105**	1.26**	0.0475**	409.97**	0.569**	2.36**	19.1949**
Grafting	1	0.0021**	0.15**	0.0094**	80.20**	0.194**	0.878**	3.3575**
Salt stress × humic acid	4	0.00080**	0.099**	0.0046**	16.16**	0.109**	0.274**	0.1220**
Salt stress × grafting	2	0.00011**	0.0144*	0.001**	4.91**	0.060**	0.046**	0.0739**
Humic acid × grafting	2	0.000098**	0.0096**	0.0026**	0.82^ns^	0.051**	0.160**	0.0844**
Salt stress × humic acid × grafting	4	0.000095**	0.0077**	0.00133**	2.30**	0.022**	0.1003**	0.0144**
Error	36	0.000021	0.0041	0.000079	0.5890	0.0044	0.0233	0.037
C.V (%)	–	2.72	3.02	1.63	2.09	4.98	5.30	1.77

*ns* non-significant effect; *significant effect at the 0.05 level and **significant effect at the 0.01 level.

**Table 11:** Effect of the different levels of salt stress, humic acid and grafting on cucumber leaf cinnamic acid, p-coumaric acid, caffeic acid, cerulic acid, Saponin, phenylalanine ammonia lyase (PAL) and polyphenol oxidase (PPO).

Salt stress (dS m^−1^)	Humic acid (mg L^−1^)	Grafting	Cinnamic acid (µg g^−1^ FW)	p-Coumaric acid (µg g^−1^ FW)	Caffeic acid (µg g^−1^ FW)	Ferulic acid (µg g^−1^ FW)	Saponin (mg g^−1^)	PAL activity (Unit mg^−1^ protein)	PPO (Unit mg^−1^ protein)
0	0	Ungrafting	0.070 ± 0.00^m^	1.01 ± 0.02^k^	0.40 ± 0.00^m^	0.80 ± 0.06^k^	20.18 ± 0.29^p^	1.00 ± 0.02^k^	7.03 ± 0.08^o^
		Grafting	0.080 ± 0.00^m^	1.07 ± 0.06^k^	0.40 ± 0.00^m^	0.84 ± 0.05^k^	22.03 ± 0.63^o^	1.68 ± 0.06^j^	7.31 ± 0.08^no^
	100	Ungrafting	0.089 ± 0.00^l^	1.30 ± 0.01^j^	0.41 ± 0.00^m^	0.81 ± 0.01^k^	24.07 ± 0.59^n^	2.04 ± 0.00^i^	7.51 ± 0.11^n^
		Grafting	0.100 ± 0.00^k^	1.46 ± 0.01^i^	0.43 ± 0.01^l^	0.85 ± 0.01^jk^	26.11 ± 0.61^m^	2.04 ± 0.03^i^	8.03 ± 0.08^m^
	200	Ungrafting	0.104 ± 0.00^jk^	1.67 ± 0.00^h^	0.46 ± 0.00^k^	0.88 ± 0.00^ijk^	28.14 ± 1.17^l^	2.11 ± 0.05^hi^	8.83 ± 0.11^l^
		Grafting	0.109 ± 0.00^j^	1.95 ± 0.04^g^	0.47 ± 0.00^j^	0.90 ± 0.11^ijk^	30.18 ± 0.89^k^	2.36 ± 0.02^h^	9.30 ± 0.23^k^
5	0	Ungrafting	0.110 ± 0.00^i^	2.02 ± 0.00^fg^	0.50 ± 0.00^i^	0.90 ± 0.00^ijk^	31.25 ± 0.22^k^	2.68 ± 0.06^g^	9.78 ± 0.10^j^
		Grafting	0.130 ± 0.00^h^	2.04 ± 0.00^fg^	0.50 ± 0.00^i^	0.95 ± 0.01^hij^	33.33 ± 0.29^j^	2.64 ± 0.05^f^	10.02 ± 0.04^j^
	100	Ungrafting	0.160 ± 0.00^g^	2.07 ± 0.00^f^	0.50 ± 0.00^i^	1.00 ± 0.00^hi^	35.03 ± 0.03^i^	3.01 ± 0.00^ef^	10.50 ± 0.11^i^
		Grafting	0.180 ± 0.00^f^	2.11 ± 0.02^f^	0.52 ± 0.00^h^	1.04 ± 0.01^h^	37.07 ± 0.06^h^	3.04 ± 0.00^ef^	11.03 ± 0.08^h^
	200	Ungrafting	0.190 ± 0.00^e^	2.24 ± 0.02^e^	0.55 ± 0.00^g^	1.16 ± 0.07^g^	39.11 ± 0.09^g^	3.08 ± 0.00^ef^	11.60 ± 0.11^g^
		Grafting	0.200 ± 0.00^d^	2.35 ± 0.03^de^	0.58 ± 0.00^f^	1.36 ± 0.02^f^	40.11 ± 0.09f^g^	3.19 ± 0.02^ef^	12.11 ± 0.07^f^
10	0	Ungrafting	0.210 ± 0.00^d^	2.44 ± 0.01^d^	0.60 ± 0.00^e^	1.73 ± 0.00^e^	41.46 ± 0.16^f^	3.27 ± 0.00^de^	12.34 ± 0.03^f^
		Grafting	0.240 ± 0.00^c^	2.62 ± 0.01^c^	0.60 ± 0.00^e^	1.92 ± 0.00^d^	44.27 ± 0.03^e^	3.49 ± 0.34^cd^	12.95 ± 0.01^e^
	100	Ungrafting	0.250 ± 0.00^c^	2.80 ± 0.10^b^	0.64 ± 0.00^d^	2.03 ± 0.00^cd^	47.35 ± 0.06^d^	3.62 ± 0.00^c^	13.50 ± 0.10^d^
		Grafting	0.250 ± 0.00^c^	2.85 ± 0.01^b^	0.66 ± 0.00^c^	2.08 ± 0.00^bc^	49.90 ± 0.09^c^	3.68 ± 0.00^c^	13.96 ± 0.08^c^
	200	Ungrafting	0.260 ± 0.00^b^	3.01 ± 0.06^a^	0.70 ± 0.00^b^	2.18 ± 0.01^b^	53.00 ± 0.12^b^	3.96 ± 0.04^b^	14.50 ± 0.22^b^
		Grafting	0.280 ± 0.00^a^	3.10 ± 0.02^a^	0.82 ± 0.00^a^	2.70 ± 0.00^a^	58.66 ± 0.16^a^	4.65 ± 0.06^a^	15.35 ± 0.05^a^

In each column means that a common letters are significantly different at the 5% level (Duncan's multiple range tests). Values express mean ± SE (n = 3).

#### Phenylalanine ammonia lyase (PAL) and polyphenol oxidase (PPO) activities

In all three levels of NaCl, the phenolic enzymes in grafted cucumber increased with increasing humic acid concentration. The highest concentration of PAL (4.65-unit mg^−1^ protein) and PPO (15.35-unit mg^−1^ protein) enzymes was observed at the maximum salinity level (10 dS m^−1^ NaCl) and humic acid application (200 mg L^−1^) in grafted cucumber (Table 11).

### Leaf mineral concentrations

Salinity stress, humic acid, grafting and interaction between salinity stress and humic acid had a significant effect (p ≤ 0.01) on root and leaf K^+^, Na^+^, and Ca^2+^ and Cl^−^ concentration in cucumber plants (Table 12). According to the obtained results (Table 13), K^+^ and Ca^2+^ levels of root and leaf of both grafted and ungrafted cucumber plants decreased with increasing salinity level. However, increasing the level of humic acid from 0 to 200 mg L^−1^ in both grafted and ungrafted cucumber plants under salinity stress, the concentration of K^+^ and Ca^2+^ increased. Humic acid, in contrast to salinity, decreased Na^+^ and Cl^−^ content of root and leaf of grafted and ungrafted cucumber. The highest amounts of Na^+^ and Cl^−^ in root and leaf were observed in the treatment of 10 dS m^−1^ along with 0 mg L^−1^ of humic acid. On the other hand, the highest amount of K^+^ and Ca^2+^ in cucumber root and leaf was found in the treatment of 0 dS m^−1^ NaCl and 200 mg L^−1^ of humic acid (Table 13).

**Table 12:** Results of analysis of variance effect different levels of salt stress, humic acid and grafting on root and leaf K^+^, Na^+^, and Ca^2+^ and Cl^−^ concentration in cucumber plants.

Means of square									
S.O.V	df	Leaf K^+^	Root K^+^	Leaf Na^+^	Root Na^+^	Leaf Ca^2+^	Root Ca^2+^	Leaf Cl^−^	Root Cl^−^
Salt stress	2	951.76**	405.13**	331.90**	671.00**	2867.94**	73.95**	5107.35**	1543.01**
Humic acid	2	98.58**	47.69**	61.47**	72.56**	400.435**	10.05**	821.39**	216.30**
Grafting	1	24.99**	8.16**	12.56**	9.31**	111.94**	1.92**	227.34**	32.13**
Salt stress × humic acid	4	10.05**	1.19**	8.36**	3.85**	136.52**	0.19**	24.24*	12.65**
Salt stress × grafting	2	4.61^ns^	0.22^ns^	2.69^ns^	0.08^ns^	32.56^ns^	0.07^ns^	11.77^ns^	1.85^ns^
Humic acid × grafting	2	0.24^ns^	0.003^ns^	1.13^ns^	0.16^ns^	5.68^ns^	0.04^ns^	6.35^ns^	0.93^ns^
Salt stress × humic acid × grafting	4	0.27^ns^	0.05^ns^	1.50^ns^	0.73^ns^	5.05^ns^	0.02^ns^	23.00*	0.47^ns^
Error	36	0.71	0.15	0.35	1.48	2.70	0.01	6.91	1.76
C.V (%)	–	3.96	2.39	10.89	11.09	4.83	1.36	5.95	

*ns* non-significant effect; *significant effect at the 0.05 level and **significant effect at the 0.01 level.

**Table 13:** Mean comparison of the effect of different levels of salt stress and humic acid on root and leaf K^+^, Na^+^, and Ca^2+^ and Cl^−^ concentration in cucumber plants.

Salt stress (dS m^−1^)	Humic acid (mg L^−1^)	Leaf K^+^ (g kg^−1^ DW)	Root K^+^ (g kg^−1^ DW)	Leaf Na^+^ (g kg^−1^ DW)	Root Na^+^ (g kg^−1^ DW)	Leaf Ca^2+^ (g kg^−1^ DW)	Root Ca^2+^ (g kg^−1^ DW)	Leaf Cl^−^ (g kg^−1^ DW)	Root Cl^−^ (g kg^−1^ DW)
0	0	25.45 ± 0.44^c^	19.21 ± 0.26^c^	2.88 ± 0.07^f^	7.35 ± 0.36^f^	37.96 ± 1.45^c^	10.90 ± 0.09^c^	34.26 ± 0.76^e^	33.46 ± 0.25^g^
	100	29.23 ± 0.69^b^	21.20 ± 0.22^b^	0.63 ± 0.27^g^	4.04 ± 0.30^g^	47.96 ± 0.88^b^	11.66 ± 0.11^b^	29.11 ± 1.12^f^	30.82 ± 0.59^h^
	200	32.98 ± 0.98^a^	23.21 ± 0.27^a^	0.21 ± 0.02^g^	1.99 ± 0.13^h^	58.23 ± 2.42^a^	12.56 ± 0.10^a^	18.40 ± 2.82^g^	24.13 ± 0.86^i^
5	0	17.75 ± 0.19^e^	14.83 ± 0.10^f^	6.33 ± 0.17^cd^	13.06 ± 0.14^cd^	27.50 ± 0.74^e^	9.30 ± 0.08^f^	51.31 ± 0.71^c^	43.16 ± 0.36^d^
	100	19.08 ± 0.22^e^	15.81 ± 0.13^e^	5.59 ± 0.12^d^	12.10 ± 0.11^de^	31.11 ± 0.25^d^	9.83 ± 0.04^e^	42.63 ± 0.98^d^	39.31 ± 0.49^e^
	200	21.56 ± 0.35^d^	17.11 ± 0.19^d^	4.19 ± 0.25^e^	10.71 ± 0.38^e^	33.11 ± 0.36^d^	10.38 ± 0.06^d^	38.78 ± 0.36^d^	36.06 ± 0.25^f^
10	0	13.59 ± 0.23^g^	9.96 ± 0.34^i^	13.34 ± 0.97^a^	18.77 ± 1.23^a^	22.27 ± 0.45^f^	6.78 ± 0.16^i^	67.76 ± 2.74^a^	49.91 ± 0.26^a^
	100	15.19 ± 0.09^f^	11.81 ± 0.22^h^	8.80 ± 0.31^b^	16.44 ± 0.35^b^	23.67 ± 0.13^f^	7.68 ± 0.06^h^	59.38 ± 0.37^b^	48.28 ± 0.19^b^
	200	16.25 ± 0.17^f^	13.45 ± 0.15^g^	7.35 ± 0.13^c^	14.46 ± 0.18^c^	24.67 ± 0.23^ef^	8.51 ± 0.12^g^	55.70 ± 0.46^b^	45.70 ± 0.36^c^

In each column means that a common letters are significantly different at the5% level (Duncan's multiple range tests). Values express mean ± SE (n = 3).

## Discussion

The disturbances in the physiology and biochemistry of plants subjected to environmental stresses can be detrimental for plant growth productivity^46^. In the present study, several parameters of growth and yield components (plant height, stem and root fresh and dry weight, number of leaf, fresh weight of fruit, number of fruit and fruit yield) decreased significantly with increasing salinity level in both grafted and ungrafted cucumber, with the most pronounced negative effect found at the highest level (10 dS m^−1^). These results are consistent with the findings of Sardar et al.^47^ in lettuce (*Lactuca sativa* L.). The elevated concentration of Na^+^ and Cl^−1^ in the soil solution of saline soils compared to all other elements, is responsible for the disruption in nutrient uptake and transport to the aerial plant parts and concomitantly plant growth and yield reduction. Another reason for the growth reduction is the inhibitory effect of salinity stress on the absorption and transport of photosynthetic substances along with the decrease in photosynthesis and photosynthetic pigments (chlorophyll *a* and chlorophyll *b*)^48^.

Vegetable grafting emerges as an effective technique for enhancing salt tolerance^28^. Some rootstocks, mainly hybrids for tomato, melon, pepper and cucumber, have demonstrated resilience to salinity^4,49–52^. According to the results of this research, salinity has reduced the fresh and dry weight of both grafted and ungrafted cucumber plants (Table 2). This can be attributed to reduced cell division, ionic imbalance, reduced water absorption, impaired absorption of elements, the effect of toxic ions, especially Na^+^, impaired absorption, regeneration and metabolism of nitrogen and protein, stomata closure and reduced photosynthetic efficiency^53^. The impact of salinity in root growth is evident in the present study, leading to a reduced capacity to uptake and transport water and nutrients from the soil to the aerial parts of both grafted and ungrafted cucumber. Several reports corroborate the decrease in dry matter production due to the increased Na^+^ concentration in plants^54,55^. In the present study, the disruption of the balance of nutritional elements and the delay in morphological, metabolic and genetic processes, are the important reasons for the observed reduction in the growth of ungrafted cucumber, especially at the salinity level^56^ of 10 dS m^−1^.

This study showed that foliar application of humic acid reduced the negative effect of salinity on cucumber growth and yield. Humic acid, known for increasing nutrient uptake and plant growth through hormonal effects, metabolic alterations, and chelating properties can counteract the negative effects of salinity^57^. For plant height increase through humic acid application a mechanism similar to gibberellin-like compounds is considered responsible. By affecting H^+^-ATPase activity of roots and distribution of root nitrate, humic acid leads to changes in the distribution of cytokinins, polyamines, and ATP, ultimately promoting stem growth^57^. By increasing nitrogen absorption, humic acid can cause an increase in various proteins, especially enzymes and proteins participating in the photosynthesis cycle, such as cytochromes, ferredoxins, plastocyanin, and Rubisco enzyme, thereby improving the vegetative growth of plants^58^. The impact of the application of humic acid on plant physiology and soil physical, chemical and biological properties is imperative in increasing tolerance to environmental stress^59^. The positive impact of the application of humic acid on plant growth and soil chemical properties under salinity stress conditions have been reported for quinoa (*Chenopodium quinoa* Willd L.)^60^. In the research conducted by Al-Zubaidi et al.^61^, unlike salinity, humic acid increased the amount of leaf surface and dry matter of cauliflower (*Brassica oleracea* var. botrytis). Humic acid not only stimulates plant growth, but has a more pronounced effect on root growth, by increasing root volume and expanding root system, thereby leading to increased water uptake^62^. This is particular important under salt stress conditions where root growth is affected. In the corn (*Zea mays* L.), the application of humic acid under salt stress conditions caused a significant increase in fresh and dry weight of plant and root as well as membrane stability^63^. In addition, humic acid can alleviate the negative effect of salinity stress on plant growth by reducing Na^+^ uptake^64^. Therefore, it is possible that in the present study, humic acid has provided suitable conditions for better vegetative growth of cucumber under salt stress conditions by increasing the absorption of nutrients and chelating Na^+^ ions. According to the research conducted by Guo et al.^52^, the use of luffa (*Luffa cylindrica* Roem., cv. Cuixiuhua) rootstock for cucumber under salt stress conditions reduces the transfer of Na^+^ to aerial organs and improves the yield and quality of grafted cucumber. In the present study, the positive effect of grafting and foliar application on the growth characteristics and yield of cucumber under salt stress conditions is evident. Indeed, in the present study, cucumber grafted plants have a more vigor and robust root system, resulting in a higher water and nutrient uptake rate, and higher net assimilation rate of CO_2_ compared ungrafted plants^30^. These results provide further support for the hypothesis that the yield of cucumber plants subjected to salinity stress could be improved by grafting.

According to the obtained results (Table 6), salt stress reduced the amount of photosynthetic pigments. The reduction in the contents of chlorophyll *a* is a result of the increase in the salinity levels, increasing the activity of the chlorophyllase enzyme and being related to the reduction in the number of chloroplasts, affecting the thylakoid membranes and constituting a recurrent symptom of oxidative stress. The reduction in the chlorophyll contents is related to lipid peroxidation and the increase in the generation of ROS. This attributed to the increase of ROS in chloroplast, which causes the destruction of the chloroplast membrane and the separation of the phytol tail from the porphyrin ring of chlorophyll^65^. Salinity stress can also increase the amount of chlorophyllase enzyme and stress hormones such as abscisic acid and ethylene further reducing the amount of chlorophyll^66^. Additionally, competition of glutamine kinase enzyme with glutamate ligase enzyme, can cause more consumption of glutamate (i.e. precursor of chlorophyll and proline) in proline production pathways, limiting chlorophyll biosynthesis^67^. In contrast, humic acid has a positive effect on the levels of photosynthetic pigments by increasing chlorophyll synthesis and delaying its degradation. Also, humic acid ability to increase nutrient uptake and its cytokinin-like properties are responsible for chlorophyll preservation and delay in leaf senescence. As a result, it can be said that in the present research, applying humic acid may lead to minimizing the chlorophyll decay and boosting the leaf chlorophyll content under salinity conditions by increasing the cell membrane stability and boosting the absorption of nutrients such as nitrogen which is related to the chlorophyll synthesis^55,68^, thereby increasing vegetative growth and yield of cucumber in the present study and soybean (*Glycine max* L.) in the study of Maıwan et al.^69^.

Under salinity stress conditions, the increase of proline serves as a defense mechanism, playing a significant role in maintaining membrane structure and osmotic regulation. In addition, due to its hydrophilic nature, proline may replace water molecules around nucleic acids, proteins, and membrane molecules, thereby mitigating the damaging the effects of ions on these compounds^70^. Another function of proline is to protect the plasma membrane scavenging hydroxyl radicals and active oxygen^71^. By increasing the amount of compatible osmolytes such as proline, humic acid contributes to osmotic regulation in plant cells under stress^12^. In addition, humic acid increases the nitrogen uptake by plants, leading to increase in free amino acids, including proline^72^. In agreement with the results of the present study, Bano et al.^73^ found that humic acid foliar application resulted in higher chlorophyll, antioxidant enzymes, proline and total soluble carbohydrates content of *Urochondra setulosa* grown under salt stress conditions. In the present study grafting resulted in increased amount of proline under salt stress conditions. Indeed, grafting onto specific rootstocks, such as the *Cucurbita maxima* × *Cucurbita moschata* interspecific hybrid rootstocks VSS-61 F_1_ and Ferro, can lead to increased total marketable yield, chlorophyll content, CAT activity and proline^30^.

According to the results of the present research, increasing the level of salinity and humic acid showed an increase in the amount of total soluble carbohydrates in grafted and ungrafted cucumber compared to the control. In the conditions of salt stress, the increase in the amount of total solution carbohydrates is due to the cellular osmotic regulation, and the control of the osmotic and water potential within the cell^74^. Moreover, total soluble carbohydrates play an important role in chelating Na^+^ ions^75^. The increase in the amount of total soluble carbohydrates is a result of the destruction and hydrolysis of larger molecules such as starch, converting them into sugar compounds such as sucrose and eventually into smaller molecules like glucose and fructose. This process contributes to osmotic regulation and increases salt stress resilience^76^. An increase in the amount of total soluble carbohydrates under salt stress conditions has been reported in fennel (*Foeniculum vulgare* L.)^67^. In the present study, grafted plants exhibited a higher amount of total soluble carbohydrates than ungrafted plants under salt stress conditions, consistent the findings in grafted tomatoes on the commercial hybrid (cv. Bark) and eight wild tomatoes^49^. Humic acid has hormone-like activity and absorption of mineral elements such as phosphorus and increases in plants, this can improve the total soluble carbohydrates in plant cells^73^.

In the present study, increasing salinity level and humic acid led to increase in the amount of total soluble protein in both grafted and ungrafted cucumber aligning with the finding on coriander (*Coriandrum sativum* L.)^56^, wheat (*Triticum aestivum* L.)^77^ and olive (*Olea europaea* L.)^78^. This positive effect of humic acid application on total soluble protein can be attributed to the increased nitrogen uptake concentration and the pseudo-hormonal properties of cytokinin and preventing interruptions in the activity of enzymes^29^. The increase in total soluble protein in grafted cucumbers under salt stress conditions is consisted with the findings in grafted peppers subjected to salt stress^28^.

The production of free radicals in stressful conditions can damage cell components as membrane lipids, proteins and nucleic acids^79^. Severe stresses, can cause parts of the bilayer phospholipids of the membrane to become hexagonal and transform the membrane structure into a porous state resulting in material leakage^80^. In the present study, EL increased significantly with increasing salinity level. In contrary but the application of humic acid at a concentration of 200 mg L^−1^ in grafted and ungrafted cucumber caused a significant decrease in EL by elevating the antioxidant defense system and concomitantly mitigating the adverse effects of salinity stress^81^. The reduction in electrolyte leakage in the leaf blade of cucumber plants can be explained by the protection of the cell membrane and the photosynthetic activity, as humic acid interacts with the signaling of ROS, reducing oxidative stress. Similar results were also obtained for bean (*Phaseolus vulgaris* L.), where foliar application of humic acid reduced EL under salinity stress and improved RWC^82^.

Indeed, antioxidant enzymes such as CAT and POX and phenolic compounds play an important role in absorbing and neutralizing free radicals^83^ and preventing damage to lipids, proteins and nucleic acids^84^. According to the results of the present research, the activity of CAT and POX enzymes and total phenol and flavonoid increased under salt stress, reaching the highest levels at 10 dS m^−1^ NaCl, thereby indicating their defense role against this stress^81,85^. Moreover, foliar application of humic acid resulted in increased activity of enzymes and antioxidant compounds, similar to was found in rice (*Oryza sativa* L.)^86^. Humic acid, through various functional groups like phenols and carboxylic acid, chelates metals, inhibits antioxidant properties, and scavenge ROS^87^. It is possible that this increase in the activity of antioxidant enzymes is due to the functional role of humic acid as an antioxidant and auxin activator or its ability as a scavenger of active oxygen species^87^. Contrary to the results of the present research, humic acid application resulted in decreased total phenol and flavonoid contenting rice plants under salt stress conditions^83^. In case of grafted cucumbers, the amount of total phenol and flavonoid was higher compared to ungrafted plants.

Relative water content is an informative indicator of plant water status. The RWC reduction as a function of the increase in water salinity can be explained by the osmotic effect, which restricts water uptake by plants and affects their water potential. High RWC denotes for plant cell maintenance and continuing growth^88^. Under salinity stress, water absorption is disrupted and leaf Na^+^ concentration is increased, both leading to reduced RWC^89,90^, as in the case of the present study. Humic acid is known to increase RWC under salinity stress conditions, also evident in the present study and cauliflower (*Brassica oleracea* var. Botrytis)^91^, by reducing water evaporation through extensive binding with water molecules and promoting root K^+^ uptake^92^. The use of fig leaf gourd as a rootstock for cucumber under salinity stress, showed a positive effect on water absorption by the roots, leading to an increase in RWC. In grafted cucumbers, the reduction of ion leakage and increase in RWC under salt stress are indicators of elevated stress resilience^30^.

In the present study, exogenous application of humic acid under stress conditions had a positive effect on the amount of secondary metabolites similar to physios (*Physalis alkekengi* L.)^92^. These include phenolic acids, flavonoids, alkaloids and saponins^93^. Phenolic acids have a wide range of biological functions in plants, such as protection against ultraviolet rays, pathogens and ROS produced under environmental stress conditions such as salt stress and aerobic metabolism^94^. Increase in secondary metabolites under salinity stress was also found in Chinese cabbage (*Brassica rapa* L. ssp. *pekinensis* (Lour.) Hanelt cv. Cantonner Witkrop^95^, pepper^96^, corn^97^ and celery^98^ (*Apium graveliens* L.). Cinnamic acid, a key phenylpropanoids with strong antioxidant properties, is produced by plants in response to stressful conditions^6^. In sweet pepper, the external application of cinnamic acid under salt stress conditions increased growth characteristics, chlorophyll, activity of antioxidant enzymes, total phenol, flavonoid, proline, K^+^ to Na^+^ ratio and decreased lipid peroxidation and Na^+^ and Cl^−^ concentration^99^. In the current research, the observed increase in the amount of cinnamic acid and other phenolic acids played an important role in increasing the growth and physiological characteristics of cucumber grown under stress and grafted onto fig leaf gourd. In terms of PAL and PPO enzymes, humic acid application resulted in increased levels in plants subjected to salinity stress and grafted onto fig leaf gourd. Both enzymes are involved in the synthesis of secondary metabolites affected by environmental stresses such as drought, salinity, high temperature, high light intensity, etc.^100^. PAL is one of the enzymes for the synthesis of phenylpropanoids and salicylic acid^101^. PPO enzyme activity increases the accumulation of phenolic acids, flavonoids and lignin^102^. Also, this enzyme catalyzes the oxidation of phenols to quinine and plays a role in regulating the level of phenolic compounds of the propanoid pathway^41^. An increase in the amount of PAL and PPO has also been reported in Moldavian balm (*Dracocephalum moldavica* L.) plants under salt stress conditions^101^.

Root and leaf K^+^ concentration of cucumber decreased with salinity stress increase while the reverse was the case for Na^+^. Salinity stress is associated with a decrease in soil water potential, leading to decreased water absorption, ionic stress, especially Na^+^ and Cl^−^, and nutrient imbalances^103,104^. In specific, under salt stress K^+^ uptake is strongly affected mainly due to the competition between Na^+^ and K^+^ for uptake by the plant^104,105^. This is associated with the similarity of ionic radii of hydrated Na^+^ and K^+^, making it is difficult to distinguish these two ions during transport through ion transport systems in the membrane^106^. In the membrane and cell wall, Na^+^ ions can easily replace Ca^2+^ ions in the binding sites, leading to reduced Ca^2+^ concentration, which results in damaging permeability and integrity^107^. The decrease in root and leaf Ca^2+^ and K^+^ concentration with increasing salinity, accompanied by an increase in Na^+^ and Cl^−^ may be attributed to the aforementioned mechanisms. Research has shown that humic acid enhances the dissolution of K^+^ by acidifying the soil and increases the availability of nutrients^108^. Also, humic acid with its pseudo-cytokinin role has a positive effect on increasing K^+^ absorption^109^. The findings of the present research showed that the application of humic acid plays an effective role in improving the absorption of elements such as Ca^2+^ and K^+^ while reducing the absorption of Na^+^ and Cl^−^ in cucumber leaf and root under salt stress conditions. Pumpkin CmoNAC1 enhances salt tolerance in grafted cucumbers and increasing the K^+^/Na^+^ ratio in grafted cucumbers under salt stress^110,111^.

## Conclusions

This research reveals that salinity adversely affects the growth and physiology of cucumber plants. Humic acid application can mitigate these negative effects by inducing physiological changes in the plants. Specifically, under 10 dS m^−1^ NaCl foliar application of humic acid led to increased levels of enzymes, compounds, and phenolic acid compared to the control. This increase suggests an enhancement in salt stress tolerance in cucumbers. Application of 200 mg L^−1^ humic acid effectively compensated for plant damage, strengthened the root system, and ultimately boosted plant yield. Moreover, the utilization of fig leaf gourd as a rootstock emerges as a practical strategy to enhance water and nutrient absorption, highlighting its significance in sustainable cucumber cultivation under challenging conditions.

## Data Availability

All data are available in the manuscript file.
